# Comparison between adherence assessments and blood glucose monitoring measures to predict A1c in type 1 diabetes

**DOI:** 10.1186/1758-5996-7-S1-A170

**Published:** 2015-11-11

**Authors:** Gabriela Heiden Teló, Martina Schaan de Souza, Thaís Sturmer Andrade, Beatriz D'Agord Schaan

**Affiliations:** 1 UFRGS-Universidade Federal do Rio Grande do Sul, Porto Alegre, Brazil

## Background

Treatment adherence is crucial in patients with diabetes; however, there is disagreement on how to measure adherence in adults with type 1 diabetes (T1D). Surveys have been validated to evaluate adherence, and several studies have demonstrated a strong correlation between frequency of blood glucose monitoring (BGM) and glycemic control.

## Objective

We conducted multivariable regression analyses to compare adherence assessments and BGM measures with regard to their ability to predict A1c in adults with T1D.

## Materials and methods

Four instruments evaluated adherence: Self-Care Inventory-Revised version (SCI-R), a self-administered survey; Diabetes Self Monitoring Profile (DSMP), a survey administered by trained researchers; a categorical (yes/no/sometimes) self-report question (“In the past month, did you take care of your diabetes as your doctor recommended?”); and a continuous adherence self-evaluation, which ranged from 0-100. BGM frequency was evaluated by self-report, BGM diary, and meter download. Glycemic control was assessed by A1c (HPLC).

## Results

Participants (N=82; 63% males) were aged 39.0±13.1 yrs. with a mean diabetes duration of 21.2±11.1 yrs.; 27% had BGM frequency >4 times/day and 39% were overweight/obese. Mean A1c was 8.9±2.2% and only 11% met the target HbA1c of <7%.The adherence assessments appeared to be interrelated (P<0.01), as well as the BGM measures (P<0.001). Among the adherence assessments, DSMP score was the strongest predictor of glycemic control (r=-0.32, P=0.004), while BGM assessed by meter download was the strongest predictor of A1c among the BGM measures (r=-40, P<0.001). Moreover, the correlation between DSMP score and BGM by meter download was the strongest identified correlation in the adherence and BGM measures (r=0.52, P<0.001). All the self-report assessments had a significant but weak correlation with glycemic control (r=-0.27,-0.28; P≤0.02). The final adjusted model identified the assessment of BGM frequency by meter download as the most robust predictor of A1c (estimate effect size=-0,58, P=0.003). Demographics and clinical characteristics did not have an impact on the adherence-glycemic control relationship (P>0.05).

## Conclusions

This study provided an opportunity to evaluate and compare adherence assessments to predict HbA1c. Although surveys like DSMP are an easy-to-use instrument to assess adherence, BGM assessment by meter download seems to have the strongest relationship with glycemic control in adults with T1D.

